# *Familias con Orgullo*: Pilot Study of a Family Intervention for Latinx Sexual Minority Youth to Prevent Drug Use, Sexual Risk Behavior, and Depressive Symptoms

**DOI:** 10.1007/s11121-024-01724-4

**Published:** 2024-09-27

**Authors:** Yannine Estrada, Alyssa Lozano, Maria I. Tapia, Alejandra Fernández, Audrey Harkness, Dalton Scott, Tae Kyoung Lee, Abir Rahman, Guillermo Prado

**Affiliations:** 1 School of Nursing and Health Studies, University of Miami, 5030 Brunson Drive, #440F, Coral Gables, FL 33146 USA; 2 Department of Public Health Sciences, Miller School of Medicine, University of Miami, Miami, FL USA; 3 Department of Health Promotion and Behavioral Sciences, UTHealth School of Public Health, Center for Pediatric Population Health, Dallas, TX USA; 4 Department of Epidemiology, Cabell-Huntington Health Department, Huntington, WV USA

**Keywords:** Latinx youth, Sexual minority, Family-based intervention, Depressive symptoms, Behavioral health

## Abstract

Families are key in the healthy development of Latinx sexual minority youth (Latinx SMY), a group that experiences behavioral, mental, and sexual health disparities. Despite this, there are no family-based interventions for Latinx SMY and their families to prevent drug use, sexual risk behaviors, and depressive symptoms. The purpose of this pilot study was to evaluate the preliminary impact (i.e., estimated effect sizes) of *Familias con Orgullo* (FcO) and examine its feasibility and acceptability among 30 Latinx SMY and their parents. Parents and adolescents were randomized to FcO or a control condition and assessed pre/post-intervention. Feasibility was measured based on session completion and effect sizes. Focus groups were conducted to evaluate intervention acceptability. Findings showed promising effects favoring FcO on parent-adolescent communication (*d* = 0.46) and parental involvement (*d* = 0.34). There were also promising effects favoring FcO on suicidal thoughts (OR = 0.75) and depression symptoms (OR = 0.69). Finally, 100% of the adolescents in FcO either continued to remain drug-free or transitioned from current use to no use (from baseline to post-intervention) compared to 74% in the control. Effect sizes for condomless sex, parental monitoring, and positive parenting were small. Session completion (above 80%) and focus group findings indicated strong feasibility and acceptability. FcO holds promise for reducing drug use and depressive symptoms and improving family functioning among Latinx SMY.

Sexual minority youth (SMY; for this study, youth between 13 and 17 years old) are faced with systemic stressors such as homonegativity, discrimination and heterosexism that drive multiple health disparities, including higher levels of drug use, sexual risk behavior, and depressive symptoms, compared to their heterosexual peers (Kann et al., 2018; Marshal et al., 2011; Ocasio et al., 2016). For example, SMY who identified as gay, bisexual, or lesbian were more likely than their heterosexual counterparts to feel sad or hopeless and have had their first drink or sexual intercourse before the age of 13 (Centers for Disease Control and Prevention, 2019). Further, gay, bisexual, and lesbian youth reported feeling sad or hopeless more than twice as often as their heterosexual peers (Centers for Disease Control and Prevention, 2019). Compared to non-Latinx White SMY, Latinx sexual minority youth (Latinx SMY) reported even higher rates of these negative health outcomes (Centers for Disease Control and Prevention, 2019). Moreover, Latinx SMY represent approximately 21% of the 3.2 million SMY between 8 and 18 in the US (Mallory et al., 2014), 60–80% of which have disclosed their sexual orientation to at least one parent (D’Augelli et al., 2005). Given the magnitude of this largely overlooked population at the confluence of multiple minority identities, addressing the social and systemic drivers of these disparate negative health outcomes is a public health priority (National Academies of Sciences, Engineering, and Medicine 2020).

## Culture and Latinx Sexual Minority Youth

The intersection of discrimination and stigma based on ethnicity and sexual minority status further compound stressors for Latinx SMY. Relative to non-Latinxs, Latinx groups tend to have more negative reactions and exhibit less tolerance towards sexual minorities, which suggests that culture has a role in the disclosure process and its aftermath (Richter et al., 2017). Adherence to traditional gender roles such as *machismo* and *marianismo* (Cauce et al., 2002) has been associated with harmful attitudes towards sexual minorities (Dillon et al., 2011; Hirai et al., 2014). Additionally, internalized homonegativity may be associated with Latinx cultural values related to masculinity (Nuñez et al., 2016). Given the influence of Latinx traditional cultural values, it is therefore no surprise that some Latinx parents are unsupportive or unaccepting of their sexual minority children, which consequently leads to elevated negative health outcomes (Gattamorta et al., 2019; Mitrani et al., 2017; Ryan et al., 2009). It is plausible that parental rejection may be due to Latinx parents’ overall negative attitudes towards homosexuality (Richter et al., 2017), religious beliefs (Diaz-Stevens, 2018; Gattamorta & Quidley-Rodriguez, 2018), traditional cultural values, and expected gender roles ubiquitously present in Latinx culture (De Santis et al., 2019). Parents may also be unaware of how to be supportive and accepting and may not know how common Latinx cultural beliefs may impact the family’s acceptance and support of SMY (Lozano et al., 2021). For example, research has shown that racially and ethnically diverse SMY report less sexual minority-specific parental support than their White counterparts (Abreu et al., 2022), an unfortunate occurrence given that lower parental support is associated with more depressive symptoms among SMY (Hall, 2018). Existing family-based interventions developed for heterosexual youth may not adequately address these intersecting cultural and sexual orientation–related stressors and, as a result, may be less efficacious or effective for Latinx SMY.

## Family Dynamics and Latinx SMY

Due in part to the heteronormative values and beliefs held by some Latinx parents, the disclosure of a minority sexual orientation may cause conflict within family systems, interfere with healthy family functioning, and result in poor health outcomes for adolescents (Richter et al., 2017; Ryan et al., 2009). As noted above, Latinx SMY often experience family rejection after coming out to parents, which is associated with reduced levels of parent-adolescent communication and family cohesion (Ryan et al., 2009). Youth disclosure initiates a transition process where parents may struggle to adjust to youth identifying as a sexual minority (Chrisler, 2017), potentially leading to poor family functioning (e.g., parental involvement, positive parenting, parent-adolescent communication, and parental monitoring). Family-level dynamics such as parental support, play a crucial role in the health of Latinx SMY through the disclosure process and beyond. Parental and family support predict the general well-being of SMY, even in the absence of other social support networks (McConnell et al., 2016; Snapp et al., 2015), while a lack of parental support following disclosure to parents has been associated with increased odds of depressive symptoms and drug use in population-based surveys (Mustanski et al., 2014; Rothman et al., 2012). Among Latinx SMY specifically, family support has been associated with fewer depressive symptoms and suicide attempts (Ryan et al., 2010). Further, some parents may not know how to be supportive of their youth, and adolescents may lack the needed skills to manage stress and negative reactions that may have reverberating psychological effects.

Similarly, parental acceptance, or lack thereof, also impacts Latinx SMY well-being. For example, parental acceptance has a protective effect on Latinx SMY by mitigating the risks they may confront (Katz-Wise et al., 2017; Przeworski & Piedra, 2020; Shilo & Savaya, 2011). Families can also be a source of taunting, bullying, and commentary about mannerisms (Finlinson et al., 2008). Stigmatizing verbal messages from family members, particularly name-calling and labeling, have been associated with low self-esteem and elevated shame (Wang, 2019). The struggle to cope with these family dynamics can lead to depressive symptoms (Muñoz-Laboy et al., 2015) and drug use (Rosario et al., 2008). The saliency of family among Latinx SMY points to the relevance of intervening at the family level and empowering parents to be change agents who positively alter health outcomes for Latinx SMY. Given the potentially deleterious impact of negative family reactions to the coming out process, it is critical to develop and evaluate family-based preventive interventions targeting adverse health risk behaviors and outcomes that Latinx SMY face. Interventions need to account for the unique experiences of Latinx SMY and their families, including the interplay of homonegativity, cultural values, family rejection/support, family functioning, and individual-level factors (e.g., internalized homonegativity).

## Family-Based Interventions and Latinx Sexual Minority Youth

A systematic review of published psychological interventions identified limited evidence-based interventions for SMY (Hobaica et al., 2018). Further, most interventions were at the individual level and excluded the family system as part of the intervention, despite a rigorous body of research that demonstrates the overall positive effects of family-based interventions on health outcomes among Latinx youth (Das et al., 2016; Van Ryzin et al., 2016). Family-based interventions present an opportunity to target multiple levels of influence on SMY mental and behavioral health outcomes.

Given the combined importance of family for Latinx SMY and the efficacy of family-based interventions for Latinx youth in general (Estrada et al., 2017; Pantin et al., 2009; Prado et al., 2012), this modality may be efficacious in preventing/reducing substance use behaviors, sexual risk behavior, and adverse mental health outcomes among Latinx SMY. Therefore, in this study, we examine one central research question: Is a family-based intervention, *Familias con Orgullo* (Families with Pride; FcO; Lozano et al., 2022), designed to prevent and reduce substance use, sexual risk behavior, and depressive symptoms among Latinx SMY feasible and acceptable to Latinx families? Further, we examined the preliminary impact of the intervention in preventing drug use, sexual risk behavior, and depressive symptoms via an examination of effect sizes. Finally, we also monitored intervention fidelity via recordings of intervention sessions.

FcO (Lozano et al., 2022) seeks to prevent drug use, sexual risk, and depressive symptoms through improvements in family functioning (defined as parent-adolescent communication, parental involvement, parental monitoring, and positive parenting). SMY are a heterogenous group with unique needs for each subgroup (e.g., cisgender gay, lesbian). For example, subgroup differences indicate more prevalent risk behaviors among bisexual youth who were not sure and among youth who have sexual contact with both sexes, compared to only same-sex contact (Rasberry et al., 2018). Nonetheless, there are also overlapping needs that cut across all sexual minority subgroups such as positive family functioning, affirmation, and support of SMY. Therefore, a broad intervention focused on these family-level factors is appropriate for all subgroups.

## Methods

This study was approved by the University of Miami Institutional Review Board, and all participants completed informed consent and assent before data collection.

FcO builds from our extensive work with *Familias Unidas* (United Families), a family-based preventive intervention for Latinx youth and their families that has successfully reduced poor health and behavioral outcomes (e.g., drug use) by targeting family functioning (Estrada et al., 2015, 2017; Pantin et al., 2009; Prado et al., 2007, 2012). Although not designed specifically for Latinx SMY, synthesized data from five Familias Unidas trials (Estrada et al., 2015, 2017; Pantin et al., 2009; Prado et al., 2007, 20012) with a small Latinx SMY subsample (*n* = 194) showed improvements in family functioning factors such as parent-adolescent communication and parental monitoring of peers, relative to a standard of care (Ocasio et al., 2022). However, no significant effects were found on drug use for Latinx SMY, which speaks to the importance of developing interventions such as FcO.

FcO consists of 14 sessions (Lozano et al., 2022): seven parent group sessions, three multi-adolescent group sessions, and four family sessions per parent-youth dyad. The intervention was delivered in person and in Spanish (with some family sessions in English) in university offices at the beginning of the study but had to be shifted to web conference delivery due to COVID-19 during the second cohort. COVID-19 also extended our recruitment timeline.

### Parent Group Sessions

The seven (2 h) parent group sessions bring parents together to foster social support and family functioning and increase parental support and acceptance for the adolescent. Through a participatory learning process, parents learn about conditions Latinx SMY face and the protective factors that are modifiable and within parental reach to protect adolescents from harmful health behaviors and negative outcomes. Further, the participatory nature of the intervention allows for discussion of the unique needs and goals that parents have for their youth and are encouraged to be agents of change on behalf of their youth.

### Adolescent Latinx SMY Group Sessions

The main goal of the adolescent group sessions is to build adolescent skills in confronting and managing stressors related to homonegativity and heterosexism. Youth learn how to foster their mental health by developing skills in emotion regulation, relaxation training, and goal setting. They also learn problem-solving skills and how to protect themselves from sexually transmitted infections and the harmful effects of drug use.

### Family Sessions

The facilitator and family participate in four (1 h) family sessions where parents transfer the competencies learned in the group sessions to their adolescents, foster supportive relationships, and increase parent-adolescent communication.

Participant recruitment, intervention delivery, post-intervention qualitative interviewing, and follow-up ran from November 2019 to February 2021, in Miami-Dade County, FL. Participants were recruited with posted flyers in community agencies that serve SMY. Additionally, adolescent participants were recruited in person from community settings that serve SMY and through referrals from participants. Families were eligible to participate if adolescents (1) were 13–17 years old; (2) were attracted to an individual of the same sex or both sexes; reported same-sex behavior or sex behavior with both sexes; and/or identified as gay, lesbian, or bisexual; (3) were of Latinx origin; and (4) had disclosed their sexual orientation to the parent participating in the study (i.e., primary caregiver). Parents had to (1) be of Latinx descent and (2) have a sexual minority child between 13 and 17 years. Anyone with a significant role in parenting the adolescent was allowed to participate in the program (e.g., grandparent, second parent) along with the eligible parent and adolescent. This inclusion criteria was broad enough to include the heterogeneity of SMY. For example, we did not require youth to identify with a specific category but rather base inclusion on attraction, same-sex behavior, or identity. The study’s exclusion criteria included (1) adolescents identified as transgender, and (2) family had plans to move out of South Florida during the study period. We decided to exclude transgender youth because from our qualitative research with Latinx SMY, which included a small number of transgender youth, we received feedback that the intervention did not have a focus on gender identity, lacked important components, and did not meet the needs of transgender youth (despite the focus on effective communication and parental support). Table 1 contains demographic information about the study sample.

**Table 1:** Participant demographics by study condition

	Overall sample (*N* = 30)	Familias con Orgullo (*n* = 19)	Control (*n* = 11)
Adolescents			
Age	15.53 (1.167)	15.37 (1.165)	15.83 (1.168)
Time in U.S			
< 3 years	1 (3.3%)	1 (5.3%)	0 (0.0%)
3–10 years	9 (30.0%)	6 (31.6%)	3 (27.3%)
> 10 years	20 (66.7%)	12 (63.2%)	8 (72.7%)
Sexual orientation			
Heterosexual			
Non-heterosexual			
Substance use (baseline)	4.71 (17.39)	6.89 (20.92)	0.11 (0.33)
Substance use (post)	7.31 (38.03	11.16 (46.95)	0.00 (0.00)
Condomless sex (baseline)	1.43 (1.90)	1.20 (1.79)	2.00 (2.83)
Condomless sex (post)	1.06 (1.77)	0.82 (1.60)	1.60 (2.19)
Depressive symptoms (baseline)	20.76 (13.39)	22.00 (14.06)	18.40 (12.39)
Depressive symptoms (post)	23.22 (12.21)	23.56 (13.28)	22.62 (10.71)
Suicidal ideation (baseline)	15 (55.6.0%)	10 (58.8%)	5 (50.0%)
Suicidal ideation (post)	16 (55.2%)	10 (52.6%)	6 (60.0%)
Suicide attempt (baseline)	4 (13.8%)	3 (15.8%)	1 (10.0%)
Suicide attempt (post)	3 (10.3%)	3 (15.8%)	1 (9.1%)
Parents			
Age	44.90 (7.581)	45.58 (7.478)	43.73 (7.976)
Time in U.S			
< 3 years	1 (3.3%)	1 (5.3%)	0 (0.0%)
3–10 years	7 (23.3%)	4 (21.1%)	3 (27.3%)
> 10 years	22 (73.3%)	14 (73.7%)	8 (72.7%)

Feasibility and acceptability of FcO were evaluated via two sequential cohorts in a randomized controlled trial with 30 parent-adolescent dyads. For each cohort, we began by randomizing participants to either FcO (*n* = 19 families) or a no-intervention control condition (*n* = 11 families) utilizing a 2:1 randomization ratio. We analyzed attendance completion rates to determine feasibility. To determine acceptability, we collected focus group data from Latinx parents and SMY that participated in the intervention. Additionally, we conducted qualitative exit focus groups after each cohort to obtain participant feedback regarding the intervention. Adolescents and parents were surveyed at baseline and post-intervention (within a week of program completion) and received $30 and $60, respectively, at each of the two survey timepoints.

## Conditions

### Familias con Orgullo

In the intervention’s 14 sessions (summarized above), FcO aims to prevent negative health risk behaviors by improving parent support for the adolescent, parent acceptance of the adolescent, family functioning (e.g., communication), and decreasing stress and sexual minority stress. To account for the various levels at which challenges for Latinx SMY occur, FcO is based on ecodevelopmental theory (Szapocznik and Coatsworth, 1999) and incorporates minority stress theory (Meyer, 2003) to conceptualize how societal factors impact SMY. Beginning with the macrosystem, FcO addresses the broad societal factors and philosophical ideals that define a particular culture and impact adolescent health, such as Latinx cultural values and norms that influence parent support and acceptance, sexual discrimination, and homonegativity. Next, the intervention focuses on the microsystem in which the adolescent participates directly and impacts family functioning, family support, and acceptance. Finally, through the adolescent and family sessions, the intervention targets adolescent individual-level factors such as cognitive, affective, and behavioral responses, including internalized homonegativity, which directly influences adolescent sexual minority stressors. The intervention targets drug use, sexual risk behavior, and depressive symptoms through establishing supportive relationships, managing minority stressors and their influence on health, and fostering positive family functioning. These theories help frame prevention intervention efforts by addressing the multiple risk and protective factors operating at multiple ecological levels of sexual minority adolescents’ intrapersonal, interpersonal, and societal/cultural contexts that influence drug use, sexual risk behavior, and depressive symptoms.

Unlike most interventions with SMY, FcO places parents in the role of change agents for the family. That is, youth outcomes are targeted through changes that the parent makes on behalf of the family and through supporting changes that youth make. For example, the parent learns how to become a support and an advocate for the youth through affirmative practices and helps the youth be a self-advocate. Through a participatory learning process, families learn how traditional Latinx cultural values (e.g., *familismo*, *machismo*, *marianismo*) may foster discrimination, minority stressors, and stigma towards Latinx SMY (Lozano et al., 2022). Group facilitators guide parents in understanding the role of culture in parenting practices and behaviors that may be stigmatizing or unsupportive (Lozano et al., 2022). Moreover, parents are provided with tools to use cultural values in a promotive way, such that they are sources of support for Latinx SMY (e.g., drawing on the importance of familial harmony in accepting youths’ sexual orientation). Further, youth learn skills to manage sexual minority stressors through emotion regulation, role plays, and the development of social support networks. Both parent and adolescent groups are 2 hours long.

### Control Condition

Families randomized to this condition did not receive an intervention from the research team. The control condition consisted of community practice/usual care with a referral. Families randomized to this condition received information and referrals regarding available services in the community that focus on supporting sexual minority youth and their families.

## Measures

All study measures were available in English and Spanish. Study data were collected with REDCap (Obeid et al., 2013). The variables examined included youth mental health (depressive symptoms and suicidality including attempts and ideation), parent and youth family functioning, adolescent drug use, and adolescent sexual risk behavior.

### Main Outcomes

Adolescent-reported depressive symptoms were measured with the *Center for Epidemiologic Studies Depression Scale* (Fendrich et al., 1990; Radloff, 1977; CES-D). Scores on the CES-D were dichotomized such that adolescents with scores greater than 16 were determined to be at risk for clinical depressive symptoms. Questions from the *Columbia-Suicide Severity Rating Scale* (Posner et al., 2011) were used to assess suicidal thoughts and attempts. Adolescent drug use was self-reported and measured using *Monitoring the Future* (Johnston et al., 2020) survey items. Adolescents were asked about cigarettes, prescription drugs, and illicit drugs. To assess adolescent sexual risk behavior, items from the *Sexual Behavior Instrument* (Jemmott et al., 1998) were used to ask adolescents about condom use during last intercourse.

### Potential Mechanisms of Effect

 Family functioning was assessed with parents and youth and operationalized with four indicators: parental involvement, positive parenting, parent-adolescent communication, and parental monitoring of peers. The *Parenting Practices Questionnaire* (Gorman-Smith et al., 1996) was used to collect information on parental involvement (*α* = 0.73) and positive parenting (*α* = 0.73). Parental monitoring of peers was assessed with the *Parent Relationship with Peer Group Scale* (Pantin, 1996; *α* = 0.72). The *Parent-Adolescent Communication Scale* (Barnes & Olson, 1985; *α* = 0.89) was used to measure communication.

### Intervention Fidelity

To monitor intervention fidelity, 100% of videotaped group sessions and 25% of randomly selected family sessions were rated by an independent fidelity rater on a 7-point scale with scores ranging from 0 (not at all) to 6 (excellent) with forms adapted from previous trials. Higher scores indicated better fidelity; content items were dichotomous and based on whether the facilitator delivered the content or not.

## Quantitative Data Analytic Plan

To examine intervention effects on continuous outcomes (e.g., family functioning indicators for parents and adolescents), we created a change score of the outcome by subtracting the baseline indicator from the same indicator at the second time point. Intervention effect sizes were calculated using Cohen’s *d* (for continuous variables; Cohen, 1988). To examine intervention effects on binary outcomes (i.e., depressive symptoms, suicidal thoughts, substance use, and condomless sex), we examined whether and to what extent endorsement of binary outcomes changed at timepoint two by condition. Then, the effect sizes of the intervention were calculated as an odds ratio (OR). Because this is a pilot study, we focus on the magnitude of the effect sizes, instead of statistical significance.

## Qualitative Procedures

Following the delivery of the intervention after each cohort, we conducted separate parent (*n* = 10) and adolescent (*n* = 10) exit focus groups, for a total of four focus groups, to collect information on acceptability (i.e., intervention content and ways to improve the intervention). Focus groups lasted approximately 60 min and were conducted in private offices (pre-COVID-19) or online (during COVID-19). Parents and adolescents were compensated for their time in completing the focus group ($80 for parents and $35 for adolescents).

Focus groups were conducted by Latinx research team members (male and female) who were either master’s level students, licensed clinical social workers, or licensed psychologists who were all trained on qualitative research methods and sensitivity training related to working with Latinx SMY. All had prior experience working with Latinx youth, SMY, and conducting qualitative research. Team members who analyzed the data agreed to openly discuss how their own lived experiences could influence their interpretation of the data and introduce bias into the results. Although the study team members who conducted the interviews and/or coded the data did not identify as lesbian, gay, bisexual, transgender, or queer (LGBTQ), the principal investigator of the study is a Latinx gay male who played a major role in developing the intervention and conceptualized the study and focus group questions.

### Qualitative Data Analysis

A general inductive approach (Thomas, 2006) was utilized to generate a summary of participant data which informed conclusions about the acceptability of the intervention. First, all study transcripts were read in detail. Next, we developed a codebook with upper-level categories for organizing data which were derived from the study objectives and lower-level subcategories which were derived directly from participant responses. Five coders were split into four different pairs to code each of the focus group transcripts. The pairs of raters independently coded their assigned transcripts in their original language (English or Spanish) using the codebook. The selected Spanish quotes were translated by native Spanish-speaking team members and translations were verified by other native Spanish-speaking team members. Raters then met to discuss codes and reached a 100% consensus on all final codes. Raters entered all final codes into Dedoose (Dedoose, 2018), a software program used to organize and categorize qualitative data.

## Results

### Quantitative Results

As noted above, due to the pilot nature of this study, we focused our quantitative analyses on effect sizes to investigate the preliminary effects of the intervention. Findings from the pilot indicate promising intervention effects for parent-adolescent communication (*d* = 0.46, 95% confidence interval (CI) [− 0.33, 1.22]), parental involvement (*d* = 0.34, 95% CI [− 0.43, 1.13]), depressive symptoms (OR = 0.69) (95% CI [0.13, 3.61]), and suicidal thoughts (OR = 0.75) (95% CI [0.15, 3.65]). Encouraging results were also found for the prevention of drug use wherein all youth randomized to FcO remained drug-free or transitioned from current use to no use (from baseline to post-intervention) compared to 74% of youth in the control condition. All these effects are considered moderate (Cohen, 1988). However, intervention effect sizes for condomless sex (OR = 1.03), parental monitoring (*d* = 0.17), and positive parenting (*d* = 0.02) were all small and not as encouraging.

A high proportion (71%) of approached families agreed to participate in the study. The average attendance for cohort one was 10.75 out of 13 sessions (*SD* = 3.10) and for cohort two 12.75 sessions out of 14 sessions (*SD* = 1.67; an additional session was added based on participant feedback during intervention development). In terms of fidelity, the average score for the family sessions was 4.63 (*SD* = 0.62) and 5.5 (*SD* = 0.7) for the group sessions*.* In terms of assessment follow-up, 100% of participants completed the post-intervention assessment.

## Qualitative Results of Post-intervention Focus Groups

Three broad themes that represent participant feedback about FcO emerged from the data: (1) potential perceived benefits to be gained from program participation, (2) interest in supporting other sexual minority youth, and (3) desire for community connection as motivation for participating. See Table 2 for participant quotes that correspond to the numbers in the parenthesis in the below text.

**Table 2:** Selected participant quotes

1. Potential perceived benefits to be gained from program participation	(1.1) “I wasn’t facing any big challenges compared to what other LGBTQ people may be facing, but my mom wasn’t as open to the subject. She was OK with me being lesbian, but she wasn’t comfortable, she wasn’t comfortable with discussing topics on it, like the sex life and all that.” (A) (1.2) “…Being in a place with the therapist and my mom, I was excited for that because I’d never been in an intimate space like that with another person and also my mom and having to talk about feelings and other stuff.” (A) (1.3) “Yes, I thought I would gain something from it. I feel like I’d be much closer to my mom, which was initially my main goal, for her to be more open-minded. From now on, when they speak of the LGBT community, she has more knowledge on it.” (A)(1.4) “With the relationship I have with my daughter, it [the program] helped both of us to grow and bond more and have better communication.” (P)
2. Interest in supporting other sexual minority youth	(2.1) So, yeah, just the idea that I could help other LGBTQs going through the same thing that I went through or people going through worse things that I went through. (A) (2.2) “To help ourselves, but it’s a study that is being done to help the people.” (P) (2.3) “When I started, when my son told me, I accepted because it seemed interesting. I told myself, ‘Well, it’s also an opportunity to meet other parents who are in the same situation…’ It may be that it helps my son more and I can also help other people, and in the same way, I would get help too.” (P)
3. Desire for community connection as motivation for participating	(3.1) “…I wanted to further educate myself on these topics because in my daily surroundings I feel like I don’t get educated enough on this. Especially at school, I feel that there’s not enough resources there to learn more about these kinds of things.” (A) (3.2) “I was really hyped, like, I was happy about meeting all these kids in the LGBT and discussing our experiences because I feel like that there aren’t that many of people to discuss our experiences with, such as coming out, our relationships with our parents, things that affect us since we’re in the LGBT. And I was really hyped about it. I was curious about how the experience would go and if it would actually help me and my mom.” (A) (3.3) “Know more about the situation. Interact with other parents also with the same situation.” (P) (3.4) “I wanted to learn more. I wanted more information. I think this is the same motivation, wanting that, that the parents and children have better understanding of ways to find…better communication, more means, and of all, what you all are doing, which is a program for the community.” (P)

*A*, adolescent; *P*, parent

### Theme 1: Potential Perceived Benefits To Be Gained from Program Participation

Adolescents noted that despite a certain degree of parental acceptance of their sexual orientation, their parents were still not comfortable discussing SMY topics prior to the intervention (1.1) and that the program offered a safe space with their parent and a professional (i.e., study interventionists) for adolescents to openly speak with parents (1.2). Adolescents thought the program could potentially provide benefits such as becoming closer to parents and helping parents be more open-minded (1.3). Parents also mirrored the sentiment of becoming closer to their adolescents as a result of the program (1.4).

### Theme 2: Interest in Supporting Other Sexual Minority Youth

Motivation for helping other sexual minority adolescents was an important driver for adolescent participation in the program (2.1). Parents also felt that the program would help not only their own family but that the study would also inform future studies that would, in turn, help other families (2.2) and that interest in the program and the opportunity to help their own family and other families motivated participation (2.3).

### Theme 3: Desire for Community Connection as Motivation for Participating

Adolescents reported a disconnect at school and in daily surroundings regarding (LGBTQ) topics and a need for further education (3.1). Additionally, the chance to meet other SMY adolescents with whom to share their experiences was appealing to adolescents (3.2). Similarly, parents were motivated to join the program to learn more about SMY and the opportunity to interact with other parents of SMY (3.3), which would allow for parental resources that facilitate communication with adolescents (3.4).

## Discussion

The current study demonstrated the feasibility, acceptability, and preliminary impact of FcO, a family intervention to prevent drug use, sexual risk behavior, and depressive symptoms among Latinx SMY. Encouraging preliminary intervention effect sizes were found for parent-adolescent communication, parental involvement, depressive symptoms, suicidal thoughts, and the prevention of drug use. However, the effect size for condom use, the measure for sexual risk behavior, was small, suggesting improvement is needed in this content area. Feasibility (i.e., session attendance) and acceptability (i.e., focus groups) analyses were also promising, suggesting FcO may be successful in a larger-scale efficacy study.

Session completion rates and focus group interviews with Latinx participants who received the intervention in the pilot study indicate that FcO is feasible and acceptable, therefore, adding to the limited body of evidence-based family-focused interventions for SMY in general and Latinx youth in particular. The high proportion (71%) of approached families who agreed to participate in this pilot, along with high session completion rates (above 80% in all cohorts), points to the feasibility of recruiting Latinx SMY and their families into preventive intervention trials. Further, the high engagement and retention rates signal the engaging nature of the intervention.

The development of FcO was based on formative work where Latinx SMY and parents were asked to provide feedback on important topics to Latinx SMY and their families in the “coming out” process and potential intervention targets (Lozano et al., 2022). Further, feedback on the content of the intervention was solicited and incorporated in multiple stages throughout the development of FcO. Although engaging participants in the research process is not new, it is often unpracticed, particularly with people from underserved, underrepresented, and marginalized groups. The lack of incorporation of participant voices is unfortunate and unaligned with research that demonstrates the benefits of including the community in the research process, including intervention adherence and improved outcomes (Green et al., 1996; Mustanski, 2011). The high engagement and participation rates in this study also provide evidence for high interest in a family-based modality with Latinx SMY. This is informative given that most interventions with SMY groups do not incorporate the family in the intervention process (Hobaica et al., 2018) despite adolescence being a critical developmental phase.

Our preliminary findings suggest that FcO shows promising intervention effects for improved family functioning (parent-adolescent communication and parental involvement), adolescent mental health (depressive symptoms and suicidal thoughts), and drug use. Specifically, the intervention affected changes in parent-adolescent communication and parental involvement, both important mechanisms in the reduction of adolescent risk behaviors and promotion of protective factors (Johns et al., 2018). Further, adolescents who received FcO were less likely to report clinically significant depressive symptoms and less likely to report suicidal thoughts. These findings are promising given the prevalence of depression and suicidality among Latinx SMY and warrant the evaluation of this intervention in a fully powered and rigorously designed efficacy study. Strong effect sizes were not found for condomless sex, parental monitoring, or positive parenting, suggesting the need for refinements to the intervention. For example, sexual risk differences among SMY show that males and females who reported sexual contact with both sexes have a higher risk of four or more sex partners and of using alcohol/drugs before sex, compared to peers who only reported same-sex sexual contact (Rasberry et al., 2018). Further, among sexual minority youth, risk behaviors are more prevalent among males and females who are uncertain of their preferences (Rasberry et al., 2018); understanding these subgroup differences is important for tailoring and aligning interventions to the specific needs of sexual minority subgroups (Rasberry et al., 2018). Importantly, administering questions to youth based on relevancy (e.g., asking about condom use only when endorsing vaginal, anal, and/or oral sex) and asking a variety of questions to evaluate multiple sexual risk behaviors and ensure that risk is being captured at various levels of sexual activity is important for future research with this intervention. Further, future work should consider parental readiness, the role of parental readiness in intervention participation, and how to reach parents who may be at different stages of acceptance or level of support.

Results from focus groups conducted with families that received FcO help to elucidate findings from our quantitative analyses. Adolescents and parents both reported perceived benefits from the intervention including the opportunity to discuss feelings, become closer to parents who perhaps were not fully comfortable with adolescents’ sexual orientation prior to the intervention, and receive resources/information pertinent to their sexual minority status. This feedback aligns well with the intervention’s goal of increasing parental support for adolescents, in addition to the promotion of positive and nurturing relationships. Additionally, the importance of advocacy emerged as a major theme, such that participants expressed that helping other SMY and their families was an important motivator for participating in the study. Relatedly, families expressed a desire for community connection as reasons for engaging and participating in the intervention.

## Limitations

This study contains limitations that should be mentioned. First, not unlike other intervention trials, there is a self-selection bias wherein participants of intervention studies may differ from non-participants. Future studies should consider strategies for engaging these families and collecting data on non-participating families. Our assessment of sexual risk behavior was limited to condom use, which excludes the differential experiences of sexual minority subgroups and is important for understanding the needs of these subpopulations. Future work should incorporate a broader examination of sexual risk that is more inclusive of the diversity of sexual experiences for sexual minority youth. The inclusion criteria required youth to have already revealed their sexual orientation to at least one parent. Therefore, 29% of families did not participate in the program due to parents that were not aware of the adolescent’s sexual orientation or were unaccepting of the adolescent’s sexual orientation. There may be significant differences between youth who have not disclosed their orientation versus those who have in terms of family functioning, drug use, sexual risk behavior, and depressive symptoms. Also, the experiences of Latinx SMY in high Latinx and SMY population areas, such as South Florida, may be fundamentally different than those in areas with less representation of these groups. This is particularly relevant now as restrictive policies (i.e., Parental Rights in Education) that directly impact SMY, and their families have been passed as law in Florida. For example, research is showing that such legislation is an additional source of stress not only for Latinx SMY but their families as well (Goldberg & Abreu, 2023) such that three-quarters of parents were worried about the law. Given the legislation, as the pilot study occurred prior to its passage, we are working on modifying some of the content in the intervention that focuses on parental investment in adolescent’s school/adolescent support systems as we prepare for our upcoming efficacy study. We have modified the content to show that although sources of support may be limited within school, there are resources for Latinx SMY outside of school from local community agencies that can provide additional supports to both Latinx SMY and their parents. Relatedly, we highlight how community agencies can be opportune locations to meet peers. This is also related to our own recruitment strategies — we will rely on community agencies for support for recruitment as we have successfully done so for this pilot study and others with Latinx SMY (Lozano et al., 2021, 2022). Additionally, in future studies, we will also recruit youth from the school system. Finally, we used family functioning heteronormative measures that have not been validated with SMY. For example, these measures may be missing components of effective parenting that are unique to SMY. There is a need to expand measures that capture the full range of SMY experiences and determinants of well-being for SMY (National Academies of Sciences, Engineering, and Medicine, 2020).

## Conclusions

There is a current lack of evidence-based preventive interventions designed to ameliorate drug use, sexual risk behavior, and depressive symptoms among Latinx SMY. Further, although the experiences of Latinx SMY extend beyond the individual level, there is a lack of empirically tested interventions designed for SMY in general, or for Latinx SMY in particular, that incorporate the family unit into prevention programming. For Latinx SMY, family relationships are important considering the high value Latinx culture places on family (Streit et al., 2018; Valdivieso-Mora et al., 2016). FcO aims to intervene at the individual and family levels to target the risk and protective factors that will optimize the development of Latinx SMY. Overall, findings from the present study indicate that the intervention was feasible and acceptable among this South Florida sample. FcO holds promise for positively impacting the health of Latinx SMY. Plans include a fully powered randomized controlled trial to determine the efficacy of FcO on reducing drug use, sexual risk behavior, and depressive symptoms among Latinx SMY.

## Data Availability

Data from this study can be made available as needed.
